# Single-stage repair of severe hypospadias using dermal graft orthoplasty and Koyanagi–Hayashi urethroplasty: a case report

**DOI:** 10.1016/j.eucr.2026.103497

**Published:** 2026-06-03

**Authors:** Yen Nguyen Le, Tuyen Tran

**Affiliations:** a Department of Pediatric Surgery, University of Medicine and Pharmacy at Ho Chi Minh City, Ho Chi Minh City, Viet Nam; b Department of Pediatrics, Vinmec Central Park International Hospital, Vinmec Healthcare System, Ho Chi Minh City, Viet Nam

**Keywords:** Hypospadias, Urethroplasty, Dermal graft, Koyanagi–Hayashi, Tunica vaginalis flap, Case report

## Abstract

Severe proximal hypospadias with marked chordee remains a reconstructive challenge, and the optimal choice between single-stage and staged repair is debated. We report a 41-month-old boy with mid-scrotal hypospadias, over 60 degrees of chordee, and penoscrotal transposition treated in a single stage by ventral dermal graft orthoplasty, Koyanagi–Hayashi urethroplasty, and tunica vaginalis flap interposition. At 26 months he had a straight penis, a glanular meatus, a normal urinary stream, and no fistula or stricture. Three independent vascular territories may mitigate the traditional graft-on-graft concern.

## 1 Introduction

Hypospadias is among the most common congenital anomalies of the male external genitalia, occurring in approximately 1 in 250 live male births.^1^ Severity spans distal forms amenable to straightforward repair to proximal variants with marked chordee and penoscrotal transposition that pose substantial reconstructive challenges.^2^^,^^3^ When ventral curvature exceeds 30°, simple degloving and dorsal plication are often insufficient, and ventral lengthening with grafting is required 4, 5, 6.

The optimal surgical approach for severe hypospadias remains controversial. Staged repair is conventionally preferred when grafting is required, based on the “graft-on-graft” concern that urethroplasty over a fresh graft bed may compromise healing.^5^^,^^6^ Single-stage repair, in contrast, reduces anesthetic exposures, cumulative cost, and the psychosocial burden of serial procedures on patients and families.^7^^,^^8^ Selection depends on anatomical severity, surgeon experience, and institutional resources.^9^^,^^10^

This report describes a single-stage configuration combining ventral dermal graft orthoplasty with Koyanagi–Hayashi urethroplasty and tunica vaginalis flap interposition. The technical rationale is that the dermal graft and the neourethra occupy distinct tissue planes with independent vascular sources, thereby mitigating the classical graft-on-graft concern. This principle parallels established strategies in hypospadias repair that emphasize multilayer vascularized coverage to reduce fistula risk, particularly the use of tunica vaginalis as an intermediate layer.^4^^,^^11^ The report adheres to the CARE guidelines (https://www.care-statement.org).

## 2 Case presentation

### 2.1 Patient and preoperative assessment

**Fig. 1:**
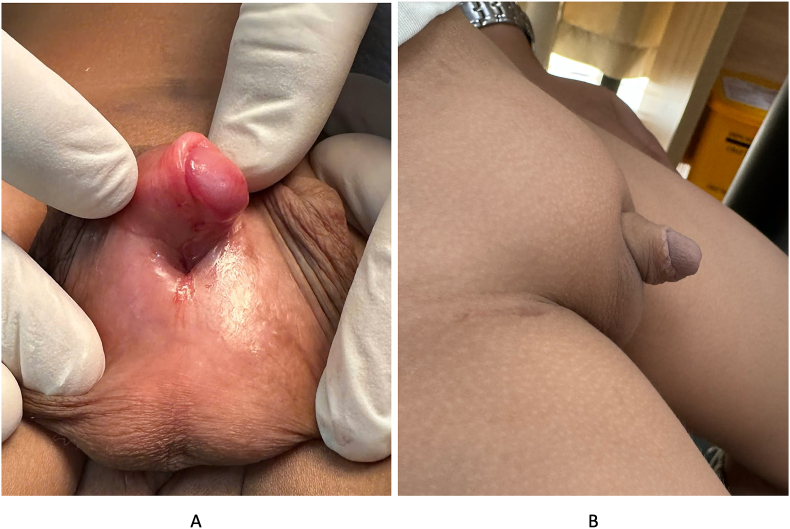
Clinical appearance. (A) Preoperative view: severe hypospadias with penoscrotal transposition. (B) Postoperative view at 26-month follow-up: a straight penis with a terminal glanular meatus.

A male infant, born April 2020 after an uncomplicated pregnancy, was diagnosed with hypospadias at birth. At 3.4 years of age he was admitted for surgical correction. Examination revealed severe penile chordee greater than 60° on artificial erection, a mid-scrotal hypospadiac meatus, and penoscrotal transposition with a bifid scrotum; he could not void in the standing position. No comorbidities were identified (Fig. 1). Stretched penile length, glans width, and formal objective scores (HOSE, GMS) were not systematically recorded; outcome assessment was therefore clinical.

After counseling on staged versus single-stage options, the family preferred minimizing the number of procedures. Given chordee >60°, dorsal plication was rejected because the expected shortening was unacceptable, and ventral lengthening with graft interposition was planned. Single-stage combined orthoplasty and urethroplasty was elected on the basis of local vascularized flap availability (Koyanagi–Hayashi rather than free graft urethroplasty^12^), an experienced surgical and anesthesia team, and dedicated postoperative single-room care. The decision was therefore not solely preference-driven but supported by favorable local tissue conditions, availability of a well-vascularized flap, and institutional experience with complex single-stage reconstructions.

### 2.2 Operative technique

**Fig. 2:**
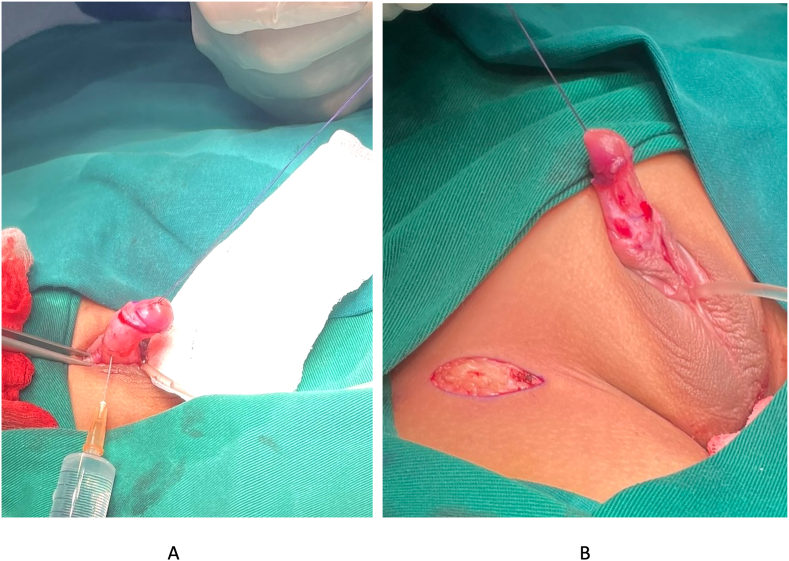
Intraoperative findings. (A) Artificial erection test demonstrating severe ventral chordee prior to correction. (B) Placement of a dermal graft for ventral tunical lengthening at the point of maximum curvature.

Under general anesthesia the incision lines were marked and the operative area was infiltrated with lidocaine containing 1:100,000 epinephrine. The penis was degloved through a circumferential subcoronal incision extended to the penoscrotal junction. An artificial erection test with normal saline confirmed persistent ventral curvature of approximately 60° at the mid-shaft (Fig. 2A).

For orthoplasty, the ventral tunica albuginea was incised transversely at the point of maximum curvature. A dermal graft approximately 15 × 25 mm was harvested from the right inguinal region, defatted, and secured to the tunical defect with interrupted 6-0 polydioxanone sutures (Fig. 2B). Repeat artificial erection confirmed adequate straightening. Anatomically, the dermal patch lies deep to the neourethra. This does not constitute a classical graft-on-graft configuration, because the overlying urethroplasty is a vascularized flap (Koyanagi–Hayashi) with its own blood supply rather than a second free graft laid on the graft bed. Dermal graft was selected over buccal mucosa or tunica vaginalis graft because of its accessible donor site, reliable take, and — critically — preservation of the tunica vaginalis for subsequent use as an interposition flap.

For urethroplasty, the Koyanagi–Hayashi technique was chosen to provide a vascularized local flap in the setting of a freshly placed ventral graft.^12^ The Koyanagi–Hayashi technique mobilizes paired parameatal-based skin flaps that retain their axial vascular pedicles; these flaps are advanced ventrally and tubularized over the urethral plate to construct the neourethra in a single stage, maintaining a blood supply independent of the underlying tunical bed. The urethral plate and parameatal skin flaps were mobilized. A 5-cm neourethra was tubularized over an 8 Fr feeding tube with continuous 7-0 polydioxanone sutures, then covered with a vascularized tunica vaginalis flap harvested from the hemiscrotum as a third tissue layer to reduce fistula risk.^11^ The catheter was exchanged for an 8 Fr Foley. Glanuloplasty was performed using interrupted 7-0 polydioxanone sutures. Penile skin was reconstructed to correct the penoscrotal transposition. The inguinal donor site was closed primarily with 5-0 rapid absorbable suture. Total operative time was approximately 3 h.

### 2.3 Postoperative management and follow-up

Oral amoxicillin/clavulanate (50 mg/kg/day) was prescribed in accordance with institutional protocol; intravenous antibiotics were not required. The patient was discharged after 24 hours. The urethral catheter remained for 10 days. Early follow-up showed satisfactory healing without infection, hematoma, or dehiscence.

At 26-month follow-up the penis was straight without recurrent chordee, and the meatus was located at the glanular tip with a vertical slit configuration. The patient voided standing with a good urinary stream. No urethrocutaneous fistula, meatal stenosis, or urethral stricture occurred. The inguinal donor site healed with a barely visible scar and the penoscrotal transposition was corrected. The family reported high satisfaction with both voiding function and genital appearance. A timeline of the clinical course is presented in Table 1.

**Table 1:** Summary of patient clinical course.

Parameter	Value
Age at Surgery	41 months 14 days
Preoperative chordee	>60°
Meatal position	Mid-scrotal
Associated anomaly	Penoscrotal transposition
Operative time	3 hours
Orthoplasty technique	Ventral lengthening with dermal graft
Urethroplasty technique	Koyanagi–Hayashi
Hospital stay	1 day
Catheter duration	10 days
Follow-up duration	26 months
Postoperative chordee	None
Final meatal position	Glanular
Complications	None
Voiding	Normal, standing position
Cosmetic outcome	Satisfactory

## 3 Discussion

The favorable outcome in this case should be interpreted cautiously and does not establish superiority of single-stage over staged repair for severe hypospadias.

Dorsal plication was rejected because, although simpler and graft-free, it shortens the penis in proportion to the degree of curvature corrected^4^^,^^5^ — unacceptable at >60° chordee given the inherently small penile dimensions often associated with severe hypospadias. Ventral lengthening with a dermal graft preserves length at the cost of technical complexity and graft-related healing concerns.^6^^,^^13^

The single-stage versus staged debate reflects genuine uncertainty in the literature. Elmoghazy et al. compared single-stage and two-stage repair in severe chordee and reported lower overall complication rates with staging.^9^ Conversely, Kumar et al. reported a 4.5% fistula rate with single-stage repair, emphasizing cost and psychosocial benefits,^7^ and Ghali et al., in 544 single-stage repairs, achieved a 96% final success rate despite a 19% initial complication rate — outcomes driven by surgeon experience and case selection.^10^ These heterogeneous outcomes suggest that the success of single-stage repair is less a function of technique selection alone and more a function of the alignment between anatomical severity, reconstructive strategy, and surgical execution.

Mechanistically, the present configuration maintains vascular separation between reconstructive layers. The dermal graft relies on imbibition and inosculation from the ventral tunica albuginea, while the Koyanagi–Hayashi flap carries an axial pedicle from parameatal tissue.^12^ The interposed tunica vaginalis flap introduces a third vascularized plane,^11^ decoupling healing zones and reducing the ischemic overlap that underlies the traditional graft-on-graft concern. Load-sharing across three independent vascular territories may partially explain the favorable healing observed.

Institutional factors also contributed: appropriate suture and catheter materials, and single-room accommodation facilitating infection control and close monitoring. These resources may not be universally available; we therefore do not advocate single-stage repair as universally superior, but as a reasonable option in selected patients at centers with the requisite infrastructure and expertise.

Limitations include the single-case design without comparative data, the absence of prospectively recorded objective parameters (HOSE, GMS, stretched penile length, glans width), and the possibility of late complications such as urethral stricture or recurrent chordee beyond the current 26-month follow-up.^14^^,^^15^ Reported intraluminal hair growth, with secondary stone formation and stricture, derives from hair-bearing skin grafts used to line the urethra in adults^16^; in the present case the graft is a de-epithelialized dermal patch placed extraluminally as a tunical reinforcement, with no contact with the urinary stream, so this mechanism does not apply, and no such complication was observed. Follow-up has not yet reached puberty; the post-pubertal behavior of a buried inguinal dermal graft under androgen stimulation therefore remains uncharacterized, and continued surveillance is warranted. Prospective comparative studies are needed to refine selection criteria for single-stage repair in severe hypospadias.

## 4 Conclusions

Single-stage repair combining ventral dermal graft orthoplasty and Koyanagi–Hayashi urethroplasty, reinforced by tunica vaginalis flap interposition, yielded a satisfactory functional and cosmetic outcome at 26 months in a boy with severe proximal hypospadias, >60° chordee, and penoscrotal transposition. While medium-term outcomes are encouraging, this single case does not establish single-stage repair as preferred; rather, it suggests that concurrent reconstruction can be considered in carefully selected patients when surgical expertise and institutional support are available. This configuration may represent a technically feasible proof-of-concept for combining graft-based orthoplasty with flap-based urethroplasty in a single stage. Longer follow-up and comparative studies are required.

## CRediT authorship contribution statement

**Yen Nguyen Le:** Writing – review & editing, Writing – original draft, Supervision, Conceptualization. **Tuyen Tran:** Writing – review & editing, Writing – original draft, Project administration, Methodology, Investigation, Data curation, Conceptualization.

## Informed consent

Written informed consent for publication of the clinical details and accompanying images was obtained from the patient's legal guardian.

## Declaration of generative AI and AI-assisted technologies in the manuscript preparation process

During the preparation of this work the authors used Claude (Anthropic) in order to assist with language editing and formatting. After using this tool, the authors reviewed and edited the content as needed and take full responsibility for the content of the published article.

## Ethics

This single-patient case report did not require formal Institutional Review Board approval under institutional policy. Written informed consent for publication of clinical details and accompanying images was obtained from the patient's legal guardian.

## Funding

This research did not receive any specific grant from funding agencies in the public, commercial, or not-for-profit sectors.
